# Comparison of the complementary feeding practices between mothers with twins and mothers with singletons

**DOI:** 10.11604/pamj.2016.24.52.8290

**Published:** 2016-05-12

**Authors:** Helena Joycelyn Bentil, Matilda Steiner-Asiedu, Anna Lartey

**Affiliations:** 1 Department of Nutrition and Food Science, University of Ghana, Ghana

**Keywords:** Infant feeding practices, complementary feeding, undernutrition

## Abstract

**Introduction:**

Several studies have been done on infant feeding practices but few have focused on twins. The aim of this study was to compare the complementary feeding practices between mothers with twins and mothers with singletons.

**Methods:**

Mother-infant pairs (50 mother-twin pairs and 50 mother-singleton pairs) with children aged 6 to 23 months were recruited from two public health clinics and communities in Tema and Ashaiman. Information was collected on the background characteristics of the mothers. Recumbent length and weight of the children were measured. Dietary information on the infants was collected using 24 hour recall. The differences between two groups were tested using independent t-student test for continuous variables and chi-square test for categorical variables.

**Results:**

The minimum dietary diversity (4+ food groups) was met by only 32% of the twins and 40% of the singletons, and 28% of the twins and 38% of the singletons met the requirement for minimum acceptable diet (minimum dietary diversity and the minimum meal frequency). Minimum meal frequency was met by 78% of the twins and 76% of the singletons. There were no significant differences between the two groups of infants. Prevalence of undernutrition was not significantly different among the two groups (twins versus singletons: underweight-26% versus 24%, stunting-20% versus 24% and wasting-14% versus 10%.

**Conclusion:**

Complementary feeding practices were suboptimal in both groups of mothers requiring interventions to improve infant feeding practices.

## Introduction

Nutrition during the first 1,000 days of a child's life can determine the difference between a promising future or one that is plagued with poor health and growth faltering. This period “window of opportunity” also indirectly shapes the future of families, communities and the stability and prosperity of the global world [1]. If children are undernourished during the critical period, they could suffer irreversible cognitive damage impacting their future health, economic wellbeing and welfare [2]. The consequences of insufficient nourishment may continue into adulthood which may affect the next generation resulting in an intergenerational cycle of growth faltering or malnutrition [3]. In the Greater Accra region alone, underweight is found among 8.3% of children between 0-5 years while 13.7% and 5.4% are stunted and wasted respectively [4]. Thus to meet the evolving nutritional needs, infants should receive timely nutritionally adequate meals with regards to quality and safety of complementary foods at the end of six months with continued breast feeding for at least 2 years of age [5, 6]. The importance of appropriate infant feeding practices during the first 2 years of life on achieving optimal health outcomes have been documented in many scientific studies [7, 8]. Some appropriate infant feeding practices include starting breastfeeding within one hour of birth, exclusive breastfeeding for the first six months and introducing appropriate and adequate complementary feeding after 6 months along with continued breastfeeding for two years or beyond. Research on most of the above mentioned infant feeding practices have primarily been focused on singletons than twins which makes one wonder what the situation is in twins. Results from a previous study on factors associated with exclusive breastfeeding of Ghanaian twins showed that mothers with twins were less likely to exclusively breastfeed and initiate breastfeeding within the first hour at birth as compared to mothers with singletons [9]. The findings from the previous study [9] prompted this current study which compared the complementary feeding practices of mothers with twins against mothers with singletons.

## Methods

Study site and subjects: the study was a cross-sectional survey conducted in the Tema Metropolis and Ashaiman Municipality; both in the Greater Accra region, Ghana. The Tema Metropolis is located along the eastern coast of Ghana whereas the Ashaiman Municipality is located about four kilometers to the North of Tema and about 30km from Accra, the capital. A random sample of 100 mother-infant pairs (50 mothers with twins and 50 mothers with singletons) aged 6 to 23 months were recruited from two public health clinics. The inclusion criteria were mothers with either twins (identical (monozygotic) or fraternal (dizygotic)) or singletons between 6-24 months of age and who reside with their biological mother. Informed consent to participate was obtained from the eligible mothers.

Data collection: in-person interviews were conducted and anthropometric measurements were taken at the clinic or at the participant home using pretested and validated questionnaires. Data on maternal characteristics such as age, education, employment status, and marital status as well as household characteristics (ownership of items, water and sanitation facilities) were collected. Mothers were asked to provide information on the child's age, gender, breastfeeding status and feeding practices. The infants’ food intake was determined using the 24 hour recall method. The mothers were asked to recall all foods given to the infants during the previous day and the number of times they fed their infants. Information from the 24 hour recall was used to determine the types of foods the infants were given the previous day and whether infants received foods from at least 4 or more food groups, and also to determine the minimum acceptable diet. The food groups were grains, roots and tubers; legumes and nuts; dairy products (milk, yogurt and cheese); flesh foods (meat, fish, poultry and liver/organ meats); eggs; vitamin-A rich fruits and vegetables; and other fruits and vegetables. Recumbent length (to the nearest 0.1 cm) and weight measurements of the children were taken. Infants’ weight was taken using a digital infant scale (Seca digital baby scale - model 334) to the nearest 0.01kg.

**Data analysis:** Data entry and analyses were done using IBM SPSS version 20. Means and standard deviations were used for continuous variables. Frequencies and percentages were used for categorical variables. The differences between the two groups were tested using independent t-student test for continuous variables and chi-square test for categorical variables. Statistical significance was set at p < 0.05. Length and weight measurements were converted to z-scores (weight-age-z scores (WAZ), weight-length-z scores (WLZ) and length-age-z scores (LAZ) using the software programme WHO Anthro Plus 2007. A z-score of < -2 standard deviation was defined as underweight (WAZ), wasting (WLZ) and stunting (LAZ), and these were compared between the twins and singletons.

**Complementary feeding practices:** Analyses of dietary diversity, meal frequency and acceptable diet were based on the new and updated infant and young child feeding (IYCF) indicators recommended by the World Health Organisation, which were based on mother's recall of foods given to her child in the previous 24 hours before the survey. The following three complementary feeding indicators were estimated from the 24 hour recall.

**Minimum dietary diversity:** Children 6 to 23 months of age who received goods from four or more of the seven food groups. The 7 food groups used for calculation of this indicator are: grains, roots and tubers; legumes and nuts; dairy products (milk, yogurt and cheese); flesh foods (meat, fish, poultry and liver/organ meats); eggs; vitamin-A rich fruits and vegetables; and other fruits and vegetables [10].

**Minimum meal frequency:** Children 6 to 23 months of age who received complementary foods the minimum number of times or more. Minimum is defined as: twice for breastfed infants 6-8 months, three times for breastfed children 9-23 months and four times for non-breastfed children 6-23 months [10].

**Minimum acceptable diet:** Children 6 to 23 months of age who had at least the minimum dietary diversity and the minimum meal frequency during the previous day [10].

**Ethical considerations:** Ethical clearance was obtained from the University of Ghana Ethics Committee for the Humanities. The study was thoroughly explained to the mothers and they were only recruited into the study after they gave their consent and signed an informed consent form.

## Results

### Socio-demographic characteristics

The socio-demographic characteristics of both infants and mothers are presented in Table 1. The two groups (twin and singleton) had similar background characteristics except for the wealth profile. There were significant differences between the two groups in terms of their wealth profile (p = 0.044). Out of the total sample of 50 singletons and 50 twins, 44% of singletons were from higher socioeconomic households, while 42% of twins were from the low socio-economic households.

**Table 1:** Socio-demographic characteristics of participants

Variable	Twin (N = 50)	Singleton (N = 50)	P value
**Child Characteristics**			
**Age (months) (Mean ± SD)**	14.66 ± 4.35	13.38 ± 5.11	0.181
**Age Group n (%)**			0.118
6-12	15(30)	24(48)	
>12-18	23(46)	14(28)	
>18-24	12(24)	12(24)	
**Sex n (%)**			0.424
Male	27(54)	23(46)	
Female	23(46)	27(54)	
**Maternal Characteristics**			
**Age (years) (Mean ± SD)**	29.38 ± 4.81	29.60 ± 5.16	0.826
**Level of Education n (%)**			0.891
No formal education	10(20)	7(14)	
Primary	6(12)	5(10)	
Junior High School	19(38)	21(42)	
Secondary	11(22)	11(22)	
Tertiary	4(8)	6(12)	
**Ethnic background n (%)**			0.385
Ga/Adangbe	4(8)	7(14)	
Ewe	19(38)	15(30)	
Akan	18(36)	23(46)	
Northern ethnicity	9(18)	5(10)	
**Marital status n (%)**			0.338
Married	46(92)	43(86)	
Single/Separated/Divorced	4(8)	7(14)	
**Employment status n(%)**			0.115
Employed	33(66)	40(80)	
Unemployed	17(34)	10(20)	
**Religion n(%)**			0.338
Christian	43(86)	46(92)	
Muslim	7(14)	4(8)	
**Wealth profile n (%)**			0.044*
High	11(22)	22(44)	
Middle	18(36)	16(32)	
Low	21(42)	12(24)	

### Dietary intake and feeding patterns

The various food groups that were consumed by the children during the previous 24 hours prior to the interview are shown in Table 2. There were no significant differences in the consumption of foods from the grains/roots/tubers, legumes/nuts, eggs, vitamin A rich fruits/vegetables and other fruit/vegetable food groups between twins and singletons. Consumption of foods from the grains/roots/tubers group was notably higher than consumption of foods from the other food groups in both groups of infants - twins (94%) and singletons (96%). In addition, the consumption of foods from the legumes and nuts group (twins - 22%, singletons - 20%), the egg group (twins - 16%, singletons - 12%) and other fruits and vegetables group (twins - 22%, singletons - 18%) were low in both twins and singletons. Very few of the mothers reported giving fruits (watermelon, pineapple, mango, banana, pawpaw, apple) to their infants. It was noted that commercial fruit drinks were given to infants in the morning as part of breakfast and in the afternoon as a snack. Mothers who did not give fruits to their infants explained that their infants refused to eat them so they stopped and some mothers also reported that they stopped giving fruits such as banana and mango to their infants because the children had diarrhea after consuming these foods. Table 3 shows the feeding schedules of the children by their mother/caregivers. The minimum dietary diversity (4+ food groups) was met by 32% twins and 40% singletons, while 78% twins and 76% singletons had the minimum meal frequency (minimum times or more) and 28% twins and 38% singletons met the criteria for minimum acceptable diet (with all 2 IYCF practices). There were no significant differences between the two groups (p > 0.05). The percentage of infants who met the minimum dietary diversity of 4 or more food groups and minimum acceptable diet was higher in singleton infants than in twins. Minimum meal frequency was met by a higher percentage of twins than singletons.

**Table 2:** Food groups consumed within 24 hours between twins and singletons

Food Groups	Twins (N = 50)	Singleton (N = 50)	P value
Grains/roots/tubers	47(94)	48(96)	0.646
Legumes and nuts	11(22)	10(20)	0.806
Dairy products	21(42)	24(48)	0.546
Flesh foods	32(64)	30(60)	0.680
Eggs	8(16)	6(12)	0.564
Vitamin A rich fruits/vegetables	23(46)	25(50)	0.689
Other fruits and vegetables	11(22)	9(18)	0.617

**Table 3:** Complementary feeding practices between twins and singletons

Complementary feeding practices	Twins (N = 50)	Singleton (N = 50)	P value
4+ food groups	16(32)	20(40)	0.405
Minimum meal frequency	39(78)	38(76)	0.812
With all 2 IYCF practices	14(28)	19(38)	0.288
Consumption of animal foods	37(74)	31(62)	0.198

### Nutritional status of children

Undernutrition was present in both groups but there were no significant differences (p > 0.05) between the two groups of children as shown in Table 4. Prevalence of underweight was 26% in twins and 24% in singletons. Stunting was 20% in twins and 24% in singletons and wasting 14% in twins and 10% in singletons.

**Table 4:** Nutritional status of children as indicated by z-scores

Anthropometry Indicator	Twins (N = 50)	Singleton (N = 50)	P value
WAZ (Mean ± SD)	-1.19 ± 1.13	-1.05 ± 1.19	0.514
LAZ (Mean ± SD)	-1.12 ± 1.12	-1.28 ± 1.31	0.399
WLZ (Mean ± SD)	-0.86 ± 1.18	-0.50 ± 1.40	0.079
**Undernutrition**	**N (%)**	**N (%)**	
Underweight	13 (26)	12 (24)	0.817
Stunting	10 (20)	12(24)	0.629
Wasting	7 (14)	5 (10)	0.538

## Discussion

At 6 months, WHO recommends that nutritionally adequate complementary foods should be introduced to the infants [11]. The results from our study showed that the foods consumed by both twins and singletons were largely from the grains, roots and tubers food group (95%) and rather low in animal source food group (62%), vitamin A rich food group (48%) and the fruits and vegetables food group (20%). This finding is consistent with evidence that the diets of Ghanaian children are typically plant-based and include little or no iron-rich animal-source foods [12] as well as findings from the IMCF project in Malawi and Cambodia [13]. Plant based diets such as porridges and other foods made from cereals, starchy roots and tubers continue to form a major part of traditional complementary foods in developing countries [14]. Unlike animal based diets which have a higher nutritional value, plant based diets are deficient in nutrients such as iron and zinc which are indispensable to the growth of a young child. The relatively high cost of animal source foods in most developing countries makes them inaccessible to many households [15] and there may also be cultural taboos against their consumption and thus result in less use for child feeding in such communities [16]. Meats, which are rich sources of critical minerals like iron and zinc as well as other essential nutrients, are often introduced only late in infancy in developed countries, and are only rarely consumed by young children [17]. Most of the mothers who were interviewed knew the importance of fruits as they were told during their antenatal visits to the hospital. Fruit drinks and artificially flavoured juices are not nutritionally equivalent to whole fruits or fruit juice and should not be included in the diets of infants and children [18]. Excessive consumption of fruit juices and drinks causes energy imbalance and contributes to diarrhoea, overweight and obesity [19] and it has been found to be a contributing factor in some children with decreased stature [20]. Results from our study showed that the overall percentage of infants who met the minimum meal frequency and consumed animal source foods were relatively high (77% and 68%, respectively), but the total percentage of infants who met the minimum dietary diversity was lower (36%) as was the total percentage of infants who met the minimum acceptable diet (33%). Sub-optimal complementary feeding practices have been previously reported in Ghana [4, 21]. The findings of Infant and Young Child Nutrition Project in Ghana showed that when infants become old enough to eat foods other than breast milk, at 6 months of age, many are not fed in a way that supports their health and growth and it further showed that only 46% of children 6-23 months of age are fed according to infant and young child feeding recommendations [12]. Minimum meal frequency was met by a higher percentage of twins than singletons. This may partly be due to the fact that twins are usually born premature or smaller at birth than their singleton counterparts, thus in an effort to promote their growth, mothers are likely to feed their twin infants more often in a day. Only 28% of the infants in our study consumed animal source foods. By 6 months, iron stores of most infants are depleted and breast milk is a relatively poor source of iron, therefore it is recommended that iron-rich foods from animal source foods should be introduced [22]. Stunting, wasting and underweight were observed in both groups of infants in the study. This was not surprising as the period of complementary feeding (6-23 months) is the time of peak incidence of growth faltering [2]. Though not significant, the prevalence of wasting and underweight were higher in twins (14% and 26%, respectively) than in singletons (10% and 24% respectively). The twin infants were found to be more likely to be wasted and underweight than singletons. This finding was not surprising. The foetal development of twins takes place in a more “crowded” womb and are born on average 3 weeks earlier than singletons [23]. As a result, they are mostly born pre-mature, small-for-gestational age and with low birth weight [24]. Low birth weight children in later life tend to have more diseases and are of poorer health status [25]. However, the effects of low birth weight are mediated by the social environment. This means that the effects are more pronounced in lower socio economic environments than in more privileged social environments. Majority of the mothers with twins in this study were from the low socio-economic household, it is therefore not surprising that they reported challenges such as lack of time, money and support/help. The mothers with twins reported that caring for their twin infants presents a “double burden” to them as compared to singletons. A number of them reported that infant foods such as “cerelac™” did not last and sometimes they would have to reduce the quantities in order to make it last for a couple more days. This practice could reduce the number of times they feed their twin infants and as a result inhibit their growth. These challenges reported by mothers with twins in this study were similar to findings by Cheng (1990) and Holditch-Davis et al., (1999) [26, 27]. They both emphasized that managing multiples is more difficult than managing singletons and it requires double the resources and efforts. These difficulties are aggravated when other siblings are in the house; meaning greater demands on parents’ efforts, time and money [27].

## Conclusion

The complementary feeding practices of mothers with twins were comparable to that of mothers with singletons. Majority of both twins and singletons were not provided with the recommended dietary diversity and acceptable diet. Educating mothers on the appropriate complementary feeding practices will help improve the number of infants who meet the recommended infant feeding guidelines.

### What is known about this topic

- Complementary feeding (6 months to 2 years) is a critical period of growth during which children are at a high risk of undernutrition due to nutrient deficiencies and illnesses;
- Nutrient deficiencies and illness during complementary feeding is due to complementary foods given too early or too late in poor nutritional quality and inadequate amounts.

### What this study adds

- The study gives information on the feeding practices of twins aged 6 months to 2 years versus singletons;
- It also provides information on under nutrition among twins versus singletons.
